# Low back pain: Influence of early MR imaging or CT on treatment and outcome - a randomized controlled study protocol

**DOI:** 10.1097/MD.0000000000024535

**Published:** 2021-03-05

**Authors:** Jianjian Zuo, Xuming Pan, Weiqiang Shen

**Affiliations:** a Bachelor of Medicine, Department of Radiology, Huzhou Traditional Chinese Medicine Hospital Affiliated to Zhejiang Chinese Medical University; b Master of Medicine, Department of Radiology, Huzhou Central Hospital& Affiliated Central Hospital of Hu Zhou University, Zhejiang, China.

**Keywords:** computed tomography, low back pain, magnetic resonance imaging, protocol

## Abstract

**Objective:**

To establish whether early use of magnetic resonance imaging (MRI) or computed tomography (CT) influences treatment and outcome of patients with low back pain.

**Methods:**

This study will be implemented from March 2021 to March 2022 at Huzhou Traditional Chinese Medicine Hospital Affiliated to Zhejiang Chinese Medical University. The experiment was granted through the Research Ethics Committee of Huzhou Traditional Chinese Medicine Hospital Affiliated to Zhejiang Chinese Medical University (R609320987). Patients who have symptomatic lumbar spine disorders at presentation are eligible for the trial if there is clinical uncertainty about the need for imaging (MRI or CT). Patients are excluded who required immediate referral for imaging (those who had signs suggestive of serious abnormalities or disease or who required surgical intervention), who have undergone MR imaging or CT of the spine within 1 year, who do not need imaging, and who have pain of a nonspinal origin. The primary outcome measure is the Aberdeen Low Back Pain (ALBP) score. Other principal outcome measure is the Short Form 36.

**Results:**

Table 1 will show the quality of life outcome measures between groups.

**Conclusion:**

This study may guide the policy makers to develop an evidence-based protocol to assess the effect of early use of MRI or CT in the treatment of patients with low back pain.

## 1 Introduction

Spinal disorders, especially low back pain, affect many people and have a negative impact on work capacity and on the overall wellbeing of an individual.^[1–3]^ Coupled with escalating health-care costs, low back pain frequently results in a significant impairment of physical and psychological health, and a decline in the performance of social responsibilities including work and family.^[4,5]^ Consequently, low back pain remains one of the most controversial and difficult conditions to manage for clinicians, patients, and policy makers. Although low back pain is highly prevalent and invades all walks of life, the consequences are especially grave for the elderly.

A global review of the prevalence of low back pain in the adult general population was published in 2012.^[6]^ Low back pain is shown to be a major problem throughout the world, with the highest prevalence among women and those aged 40 to 80 years.^[7]^ Overall, the annual prevalence of low back pain has been reported to range from 15% to 45% and the prevalence of severe low back pain continues to increase with age.^[8]^ Advanced imaging, computed tomography (CT), or magnetic resonance imaging (MRI) are helpful if radiographs are not explanatory of unremitting lower back pain or there is substantial clinical suspicion for an underlying systemic disease.^[9,10]^ MRI without contrast is generally considered the best initial test for most patients with low back pain who require advanced imaging. In patients who require advanced imaging but cannot have an MRI, a CT scan is usually the next step. It is unclear which of the diagnostic imaging pathways is most effective and cost-effective and how the imaging impacts on patient treatment. The objective of this randomized controlled protocol is to establish whether early use of MRI or CT influences treatment and outcome of patients with low back pain.

## 2 Methods

This study will be implemented from March 2021 to March 2022 at Huzhou Traditional Chinese Medicine Hospital Affiliated to Zhejiang Chinese Medical University. The experiment was granted through the Research Ethics Committee of Huzhou Traditional Chinese Medicine Hospital Affiliated to Zhejiang Chinese Medical University (R609320987) and recorded in research registry (researchregistry6420).

### 2.1 Inclusion and exclusion criteria

Patients who have symptomatic lumbar spine disorders at presentation are eligible for the trial if there is clinical uncertainty about the need for imaging (MRI or CT). Patients are excluded who required immediate referral for imaging (those who had signs suggestive of serious abnormalities or disease or who required surgical intervention), who have undergone MR imaging or CT of the spine within 1 year, who do not need imaging, and who have pain of a nonspinal origin.

### 2.2 Data collection

After we obtain informed consent, research nurses collect baseline clinical and demographic details, and participants complete health status questionnaires prior to random assignment to groups. At 12 and 24 months, health status measures and information about primary care consultation, purchase of prescription and nonprescription medicines, and discontinuation or interruption of usual activities because of low back pain are collected with postal self-completion questionnaires. Telephone calls and reminder letters are used to increase the return of questionnaires.

### 2.3 Randomization and data analysis

Sequence of random numbers is generated by a computer. Sequentially numbered sealed opaque envelopes are used for the concealment of random numbers. All the patients taking part in our experiment are randomly divided to the early imaging group (in which MRI or CT is performed as soon as was practicable) or the delayed selective imaging group (in which no MR imaging or CT is performed unless a clear clinical indication subsequently developed).

### 2.4 Outcome measures

The primary outcome measure is the Aberdeen Low Back Pain (ALBP) score. This condition-specific questionnaire allows assessment of low back pain across several dimensions, including pain, physical impairment, and functional disability.^[11]^ Responses to the 19-item questionnaire are summed and converted to a percentage score (scores range from 0 for least disabled to 100 for most disabled). Other principal outcome measure is the Short Form 36, a generic instrument that is widely used and has been shown to be a reliable and valid instrument for the assessment of functional status.

### 2.5 Statistical analysis

The analysis of all the data are conducted with the software of IBM SPSS Statistics for Windows, version 20 (IBM Corp., Armonk, NY). The data obtained are represented through the proper features, for example, standard deviation, and mean, median as well as percentage. And independent *t* tests and χ^2^-tests are respectively utilized to analyze the categorical variable and continuous variable. When *P* is less than .05, the efficacy is viewed to be statistically significant.

## 3 Result

Table 1 will show the quality of life outcome measures between groups.

**Table 1:** Comparison of clinical outcomes between 2 groups.

	Study group (n = 50)	Control group (n = 50)	*P* value
ALBP score			
Short Form 36			
Opioid consumption			
Pain score at 12 months			
Pain score at 24 months			

## 4 Discussion

Back pain affects most adults, is a leading cause of activity limitation and work disability worldwide, and is among the most common reasons for seeking healthcare.^[12,13]^ It has an enormous impact on individuals, healthcare systems, and national economies, and treatment approaches have important consequences for patients, clinicians, and society.^[14,15]^ Although most episodes are self-limiting, for a minority of patients referred to a specialist, the place of cross-sectional imaging techniques such as MRI or CT in the treatment of low back pain is controversial.^[16]^

Decisions about the use of sophisticated imaging will depend to an important extent on judgments about the value of the observed differences in outcome and whether they are worth the extra costs of early imaging. The use of MRI does not appear to affect treatment overall, and the small observed improvement in outcome is of questionable clinical importance. Although some researchers may argue that any improvement is worthwhile, given that the other costs of treatment do not appear to be increased, others may say that the cost of providing a small improvement in patients’ overall well-being is not justifiable, especially when there are competing demands for MRI resources.^[17]^ There are demands for more rigorous scientific evaluation of both the clinical effectiveness and cost-effectiveness of new health care technologies such as MRI or CT prior to their widespread diffusion and use.

## 5 Conclusion

This study may guide the policy makers to develop an evidence-based protocol to assess the effect of early use of MRI or CT in the treatment of patients with low back pain.

## Author contributions

Weiqiang Shen designs the protocol. Xuming Pan reviews the protocol and performs the data collection. Jianjian Zuo finishes the manuscript. All of the authors approved the submission.

**Conceptualization:** Jianjian Zuo.

**Formal analysis:** Xuming Pan.

**Funding acquisition:** Weiqiang Shen.

**Investigation:** Xuming Pan.

**Methodology:** Xuming Pan.

**Writing – original draft:** Jianjian Zuo.
